# Technical efficiency of public district hospitals in Bangladesh: a data envelopment analysis

**DOI:** 10.1186/s12962-019-0183-6

**Published:** 2019-07-24

**Authors:** Sayem Ahmed, Md. Zahid Hasan, Samia Laokri, Zerin Jannat, Mohammad Wahid Ahmed, Farzana Dorin, Veronica Vargas, Jahangir A. M. Khan

**Affiliations:** 1 Health Systems and Population Studies Division, icddr,b, Dhaka, 1212 Bangladesh; 2 Department of Learning, Informatics, Management and Ethics (LIME), Karolinska Institutet, SE-171 77 Stockholm, Sweden; 3 Department of Tropical Biology, Liverpool School of Tropical Medicine, Liverpool, L3 5QA UK; 4 Global Community Health and Behavioral Sciences, School of Public Health and Tropical Medicine, Tulane University, New Orleans, LA 70112 USA; 5 Department of Economics, Stockholm University, SE-106 91 Stockholm, Sweden; 6 Alberto Hurtado University, Santiago, Chile; 7 Department of International Public Health, Liverpool School of Tropical Medicine, Liverpool, L3 5QA UK; 8 The Lakshmi Mittal And Family South Asia Institute, Harvard University, Cambridge, MA 02138 USA

**Keywords:** Technical efficiency, Secondary hospitals, District hospitals, Data envelopment analysis, Bangladesh

## Abstract

**Background:**

District hospitals (DHs) provide secondary level of healthcare to a wide range of population in Bangladesh. Efficient utilization of resources in these secondary hospitals is essential for delivering health services at a lower cost. Therefore, we aimed to estimate the technical efficiency of the DHs in Bangladesh.

**Methods:**

We used input-oriented data envelopment analysis method to estimate the variable returns to scale (VRS) and constant returns to scale (CRS) technical efficiency of the DHs using data from Local Health Bulletin, 2015. In this model, we considered workforce as well as number of inpatient beds as input variables and number of inpatient, outpatient, and maternal services provided by the DHs as output variables. A Tobit regression model was applied for assessing the association of institutional and environmental characteristics with the technical efficiency scores.

**Results:**

The average scale, VRS, and CRS technical efficiency of the DHs were estimated to 85%, 92%, and 79% respectively. Population size, poverty headcount, bed occupancy ratio, administrative divisions were significantly associated with the technical efficiency of the DHs. The mean VRS and CRS technical efficiency demonstrated that the DHs, on an average, could reduce their input mix by 8% and 21% respectively while maintaining the same level of output.

**Conclusion:**

Since the average technical efficiency of the DHs was 79%, there is little scope for overall improvements in these facilities by adjusting inputs. Therefore, we recommend to invest further in the DHs for improvement of services. The Ministry of Health and Family Welfare (MoHFW) should improve the efficiency in resource allocation by setting an input-mix formula for DHs considering health and socio-economic indicators (e.g., population density, poverty, bed occupancy ratio). The formula can be designed by learning from the input mix in the more efficient DHs. The MoHFW should conduct this kind of benchmarking study regularly to assess the efficiency level of health facilities which may contribute to reduce the wastage of resources and consequently to provide more affordable and accessible public hospital care.

## Background

Sustainable development goals (SDG), developed by the United Nations General Assembly, includes health as a major component to ensure healthy lives and promote well-being for all ages by 2030 [1]. In South Asia, Bangladesh is one of the poorest and densely populated countries. The country had a population of 161 million in 2015. By 2030, this population size is expected to increase to around 218 million [2].

Constitutionally, Bangladesh government is obliged to provide basic healthcare to every citizen. To ensure healthcare for the citizens, the country has built a structured health system covering different healthcare services, health education, health promotion, and rehabilitation. The health systems have a tire structure that includes primary, secondary, and tertiary level of healthcare facilities in communities, sub-districts, districts, and divisions of the country.

The primary level health facilities include Upazila Health Complexes at the sub-districts, Union Health and Family Welfare Centers at the Unions (collection of few villages), and Community Clinics at the villages. District Hospitals or general hospitals (DHs) works as referral centers of these primary level facilities and delivers the secondary level of healthcare including treatment for non-communicable diseases and a number of other specialized cares (e.g. Cardiac, Neuroscience, and Orthopaedic hospitals). The tertiary hospitals (e.g. Medical college hospitals, specialized hospitals) of various kinds provide supports to the primary and secondary level health facilities along with specialized health services [3].

There are 62 DHs across the country and each of the districts has at least one such hospital except Rajshahi and Dhaka. In some districts, the hospitals are called ‘general hospital’ or ‘250-bed hospital’ [3]. The DHs provide primary and secondary care through the outdoor, indoor (outpatient and inpatient services), and emergency departments [4]. About 6% of the total outpatient visits and 40% of the total inpatient admissions in public facilities were served by DHs, and more than 22% of the total government health expenditure was spent through these facilities in 2015 [3, 5].

Globally, it was estimated that about USD 300 billion were being lost annually due to hospital-related inefficiency [6]. Efficiency studies are important for informed decision making to improve the performance of hospitals and reduce wastage. It is important to reduce the consumption of excessive resources in producing healthcare services for all healthcare system. Efficiency in production invariably results in better allocation of resources and increase the opportunity to serve more beneficiaries. This in turn raises important sustainability and equity implications.

In different countries (e.g. Angola, India, and Ghana) several studies were carried out in assessing the efficiency of healthcare facilities by using data envelopment analysis (DEA) [7–10]. Over the decades, DHs are playing a key role in health systems of Bangladesh as secondary hospitals. However, little has been known about the efficiency of these DHs in this context. In 1999, a study was conducted to assess the efficiency by comparing bed occupancy rate and bed turnover rate of subdistrict level healthcare facilities in Bangladesh using Lasso’s graphical model [11]. The finding suggested that large sub-district hospitals with more beds and staff were efficient and optimal. Vargas et al. 2016 assessed the efficiency of DHs using a similar approach. They found only 12 out of 62 DHs (19.4%) with greater inefficiency and around one-third of the hospital with greater efficiency and the remaining hospitals had average efficiency. However, this study only considered two indicators (e.g. bed occupancy rate and bed turnover rate) for efficiency analysis [12]. Therefore, efficiency analysis of DHs is required considering multiple input and output variables to compare their level of efficiency. Efficient DHs can contribute considerably toward achieving the health-related targets in SDG. Thus, we aimed to estimate the technical efficiency of the DHs in Bangladesh using DEA.

## Methods

We used secondary data to conduct this cross-sectional study. The efficiency scores of DHs were estimated using an input-oriented DEA  [13]. The association of institutional and environmental characteristics with the technical efficiency of the DHs was measured using a Tobit regression model.

### Sources of data

In this study, we used data from the Local Health Bulletin-2015. The bulletin consists of annual hospital service and monitoring statistics which is published annually by the Directorate General of Health Services (DGHS), Ministry of Health and Family Welfare (MoHFW), Bangladesh [3]. Population data was collected from Bangladesh Bureau of Statistics (BBS) and district wise poverty headcounts from The World Bank report [14, 15]. We selected these data sources for extracting input, output and explanatory variables for the efficiency analysis (Table 1).

**Table 1:** Selected variables and sources of information

Variables	Measuring units (in year 2015)	Source
*Input variables*		
Beds	Total number of beds available	Local health bulletin 2015 [3]
Doctors	Total number of doctors (specialists and primary care physicians) available	Local health bulletin 2015 [3]
Nurses	Total number of nurses available	Local health bulletin 2015 [3]
*Output variables*		
At least 4 ANC recipients	Total number of women received 4 ANC services	Local health bulletin 2015 [3]
Normal deliveries	Total number of normal deliveries	Local health bulletin 2015 [3]
Caesarean-section deliveries	Total number of caesarean-section service provided	Local health bulletin 2015 [3]
PNC recipients	Total number of women received PNC service	Local health bulletin 2015 [3]
Outpatient department visits	Total number of outpatient visits	Local health bulletin 2015 [3]
Inpatient admissions	Total number of inpatient admissions	Local health bulletin 2015 [3]
*Explanatory variables*		
Population size	Number of catchment population in the district where the hospital located	Bangladesh Bureau of Statistics [15]
Poverty headcount	Proportion of people living below the poverty line in the DHs area	World Bank 2010 [14]
Bed occupancy ratio	Proportion of beds occupied over a specific year	Local health bulletin 2015 [3]
Average length of stay	Average number of days patients spent in a hospital	Local health bulletin 2015 [3]
Ratio of beds to physicians	Total number of beds per physician	Local health bulletin 2015 [3]
Ratio of beds to nurses	Total number of beds per nurse	Local health bulletin 2015 [3]

### Selection of input and output variables

The selection of variables was guided by a literature review on the efficiency analysis and considering the availability of the relevant data [3, 7–10]. Inputs in hospital production are classified as labour, capital and supplies. The labour input can be disaggregated into the various professional groups such as physician, nurse, and administrative staff. Number of hospital beds can be considered as the proxy of capital [16, 17]. Due to the constraint in data we have used labor (doctors and nurses) and capital (as proxied by number of beds) as the inputs in production of hospital services.

There are two thoughts regarding the measurement of the output of a healthcare organization i.e. process approach (output of a healthcare organisation consists of intermediate services such as number of tests performed or patients served or patient days etc.) and outcome approach (consider health status as ultimate outcome) [18]. In reality, measuring processes or intermediated outcome (services) in healthcare is easier than measuring changes in health status. Further, changes in health outcome cannot be entirely attributed to healthcare as health is multi-dimensional and affected significantly by a host of other socioeconomic factors. Thus, we selected at least 4th antenatal care (ANC) recipients, normal deliveries, caesarean (C)-section deliveries, post-natal care (PNC) recipients, outpatient department (OPD) visits, and inpatient admissions as output variables in DEA.

### Explanatory variables

The explanatory variables for a Tobit regression were selected based on review of literature on efficiency analysis [16, 19, 20]. Factors that affect the efficiency of DHs were classified to environmental factors i.e. catchment population, administrative locations, and poverty headcount and institutional factors i.e. average length of stay (ALoS), bed occupancy ratio (BOR), ratio of beds to physicians (RoBTP), and ratio of beds to nurse (RoBTN) (Table 1).

### The data envelopment analysis

DEA is widely used for estimating the technical efficiency of a set of decision making units (DMUs) that accommodates multiple inputs and outputs [21]. DEA approach assumes that a set of DMUs is associated with their corresponding amount of inputs and outputs. The efficiency score is defined as a ratio of the weighted sum of the outputs to the weighted sum of the inputs [22]. It is based on a non-parametric linear programming technique which identifies an efficiency frontier on which only the efficient DMUs are placed. A DMU is considered to be technically efficient if it can produce maximum output from a given set of inputs.

In estimating the efficiency frontier, Charnes, Cooper, and Rhodes (CCR) assumed production as constant returns to scale (CRS) which means any level of increase in inputs will proportionately increase the level of output [13]. Another model proposed by Banker, Charnes, and Cooper (BCC), assumed that production as variable returns to scale (VRS) that means any increase in the level of input will either increase or decrease the level of output [23]. In the VRS assumption, a DMU may result in increasing returns to scale (IRS) or decreasing returns to scale (DRS). When output increases by a greater proportion than the increase in inputs, the production process is called IRS. On the other hand, when output increases by a smaller proportion than the increase in inputs, the production process is called DRS.

The health services production process is not linear and the VRS technical efficiency assumption in health service production may be more appropriate [10]. However, we demonstrated two types of technical efficiency, namely, CRS technical efficiency; estimated based on the CCR model and VRS technical efficiency; estimated based on the BCC model to allow the comparison of findings between two methods [13, 23]. The scale efficiency is a measure of the extent to which a DMU deviates from an optimal scale. When a DMU is operating at CRS, technical efficiency is equal to scale efficiency as CRS technical efficiency denotes that technical efficiency of a DMU cannot be attributed to deviations from optimal scale (required optimal size for given input and output mix). The scale efficiency is represented by the ratio of the scores from CRS technical efficiency and VRS technical efficiency [24].

We have utilized an input-oriented DEA model as it focuses on minimizing the use of inputs for producing the given amount of outputs. This model fits with the context of DHs since these hospitals can control over the inputs such as staffing and operating expenses or beds, rather than on how many patients get admitted or visits [25]. Our operating units or DMUs are 62 DHs for which three inputs and six outputs variables (Table 1) were selected for analysis.

### Input oriented model

The input-oriented VRS DEA model is specified as follows,

$$\begin{aligned} {\text{Eff}} & = {\text{Max}}\sum a_{r} y_{{rj_{0} }} + a_{0} \\ & \quad {\text{a}}_{\text{r}} ,{\text{ b}}_{\text{i}} \end{aligned}$$

Subject to

$$\sum a_{r} y_{rj} - \sum b_{i} x_{ij} + a_{0} \le 0;\;\forall {\text{j}}$$

$$\sum b_{i} x_{{ij_{0} }} = 1$$

$${\text{a}}_{{{\text{r}},}} {\text{b}}_{\text{i}} \ge 0;\;\forall {\text{r}},\forall {\text{i}}$$

where y_rj_ is the amount of output r produced by DH j; x_ij_ is the amount of input i used by DH j; a_r_ is the weight for output r; b_i_ is the weight for input i; n is the total number of DH, and j_0_ is the considered DH; ∀ = for all.

The efficiency score of the DHs ranges between 0 and 1. DHs that are technically efficient have a score of one or 100%, whereas, the inefficient DHs have efficiency scores of less than 1 or less than 100%.

### Tobit regression analysis

To measure the association between the inefficiency scores and number of explanatory variables we used a Tobit regression model. Since, by definition, the DEA scores range between zero and one, and some of the data tend to concentrate on these boundary values (i.e., censored for the DMUs with a value at one), ordinary least squares can not estimate the regression. For the convenience of the calculation, we assumed a censoring point at zero in this model. As a result, the efficient DHs will have score zero and the inefficient DHs will have score greater than zero. Following [26], this was performed by transforming CRS and VRS technical efficiency scores into CRS and VRS inefficiency scores and left censoring at zero as follows.

$$Inefficiency \;score = \left( {1/ Technical\;efficiency \;score} \right) - 1.$$

Both CRS and VRS technical inefficiency scores were regressed separately to estimate the association between technical efficiency scores and selected institutional and environmental characteristics (Table 1). The Tobit regression models were specified as follows,

$$Ineff_{i} = \beta_{0} + \beta_{1} POP_{i} + \beta_{2} Poverty_{i} + \beta_{3} Division_{i} + \beta_{4} ALoS_{i} + \beta_{5} BOR_{i} + \beta_{6} RoBTP_{i} + \beta_{7} RoBTN_{i} + \varepsilon_{i}$$

where Ineff is the technical inefficiency score; POP is the categorical varible of regional population (1 = if population is less than 1,000,000; 2 = if population is above 1,000,000 to 2,500,000 and 3 = above 2,500,000); Division is a categorical variable for eight different divisions (1 = Barisal, 2 = Chittagong, 3 = Dhaka, 4 = Khulna, 5 = Mymensingh, 6 = Rajshahi 7 = Rangpur and 8 = Sylhet). The ALoS, BOR, RoBTP, and RoBTN were included as continuous variable in the models. Finally, εi was the stochastic error term.

## Results

Descriptive statistics of the selected input and output variables of the DHs are shown in Table 2. The number of beds varied from 100 to 278 with a mean and standard deviation (SD) of 148 and 70 respectively. The average number of doctors (specialists and primary care physicians) and nurses was 23 and 60 respectively in the DHs. The average number of OPD visits was 149,625 patients and inpatient admission was 24,915 patients.

**Table 2:** Descriptive statistics of input and output variables

Variables	Mean	Standard deviation (SD)	Median	Minimum	Maximum
Inputs					
Number of beds	148	70	100	100	278
Doctors (specialists and primary care physicians)	23	13	19	5	60
Nurses	60	34	49	12	159
Outputs					
Number of 4 ANC recipients	1679	2811	888	–	18,548
Number of normal deliveries	1093	824	857	–	5368
Number of C-section deliveries	624	546	463	–	2756
Number of women received PNC	2607	2595	1832	66	17,493
Number of OPD visits	149,625	59,594	136,596	42,383	312,797
Number of admissions	24,915	15,612	20,964	2235	85,005

Table 3 presents the estimated CRS and VRS technical efficiency, scale efficiency, and returns to scale score of each DHs. The mean CRS technical efficiency was 79%, VRS technical efficiency was 92%, and scale efficiency was 85%. In total, 18 (29%) were CRS technically efficient among the 62 DHs. Among the remaining 44 inefficient DHs, the mean CRS technically efficiency was 70%. The Gopalganj 250 Bedded District Hospital had the lowest such efficiency (48%).

**Table 3:** Technical and scale efficiency scores and returns to scale characteristics of district hospital

Hospitals	CRS technical efficiency score	VRS technical efficiency score	Scale efficiency score	Returns to scale
Bagerhat District Hospital (BhD)	0.90	1.00	0.90	1
Bandarban District Hospital (BbD)	0.36	1.00	0.36	1
Barguna District Hospital (BuD)	0.93	1.00	0.93	1
Barisal General Hospital (BrG)	0.50	1.00	0.50	1
Bhola District Hospital (BlD)	1.00	1.00	1.00	0
Bogra 250 bed Mohammad Ali District Hospital (BgD)	0.47	0.49	0.96	1
Brahmanbaria 250 bed District Sadar Hospital (BmD)	1.00	1.00	1.00	0
Chandpur 250 bed General Hospital (CnD)	0.49	0.53	0.94	− 1
ChapaiNawabganj District Hospital (CpD)	0.97	1.00	0.97	1
Chittagong General Hospital (CtD)	0.51	0.54	0.94	− 1
Chuadanga District Hospital (CdD)	0.99	1.00	0.99	1
Comilla General Hospital (ClD)	0.59	1.00	0.59	1
Coxs Bazar 250 Bed District Sadar Hospital (CbD)	1.00	1.00	1.00	0
Dinajpur General Hospital (DnD)	0.52	0.53	0.98	− 1
Faridpur General Hospital (FdD)	1.00	1.00	1.00	0
Feni 250 bed District Sadar Hospital (FdS)	0.69	0.87	0.79	− 1
Gaibandha District Hospital (GdD)	0.84	1.00	0.84	1
Gazipur District Hospital (GiD)	0.62	1.00	0.62	1
Gopalganj 250 Bedded District Sadar Hospital (GpD)	0.46	0.48	0.97	− 1
Habiganj District Hospital (HgD)	1.00	1.00	1.00	0
Jamalpur 250 bed General Hospital (JmD)	0.63	0.72	0.87	− 1
Jessore 250 bed General Hospital (JeG)	0.79	1.00	0.79	− 1
Jhenaidah District Hospital (JdD)	1.00	1.00	1.00	0
Jholakathi District Hospital (JlD)	0.68	1.00	0.68	1
Joypurhat District Hospital (JpD)	0.74	0.80	0.93	− 1
Khagrachari District Hospital (KcD)	0.83	1.00	0.83	1
Khulna General Hospital (kuD)	0.50	0.67	0.74	1
Kishoreganj 250 bed District Sadar Hospital (KgD)	0.90	1.00	0.90	− 1
Kurigram District Hospital (KiD)	0.89	1.00	0.89	1
Kushtia 250 bed General Hospital (KsD)	0.79	1.00	0.79	− 1
Lakshmipur District Hospital (LhD)	0.93	1.00	0.93	1
Lalmonirhat District Hospital (LmD)	0.72	1.00	0.72	1
Madaripur District Hospital (MdD)	0.61	1.00	0.61	1
Magura District Hospital (MuD)	0.93	1.00	0.93	1
Manikganj District Hospital (MnD)	0.84	1.00	0.84	1
Meherpur District Hospital (MrD)	1.00	1.00	1.00	0
Moulvibazar 250 bed District Sadar Hospital (MbD)	0.54	0.60	0.90	− 1
Munshiganj District Hospital (MgD)	0.74	1.00	0.74	1
Naogaon District Hospital (NgD)	0.89	1.00	0.89	1
Narail District Hospital (NaD)	0.69	1.00	0.69	1
Narayanganj General Hospital (NyD)	1.00	1.00	1.00	0
Narsingdi District Hospital (NsD)	0.72	1.00	0.72	1
Narsingdi District Hospital (Development) (NrD)	1.00	1.00	1.00	0
Natore District Hospital (NtD)	1.00	1.00	1.00	1
Netrokona District Hospital (NkD)	1.00	1.00	1.00	0
Nilphamari District Hospital (NpD)	1.00	1.00	1.00	0
Noakhali 250 bed General Hospital (NhD)	0.65	0.67	0.96	− 1
Pabna 250 bed General Hospital (PnG)	1.00	1.00	1.00	0
Panchagarh 100 bed District Sadar Hospital (PcD)	1.00	1.00	1.00	0
Patuakhali 250 bed Sadar Hospital (PtS)	0.60	0.77	0.78	1
Pirojpur District Hospital (PrD)	0.48	1.00	0.48	1
Rajbari District Hospital (RjD)	0.78	1.00	0.78	1
Rangamati General Hospital (RgD)	0.39	1.00	0.39	1
Saidpur 50 Beded Hospital (SiD)	1.00	1.00	1.00	0
Satkhira District Hospital (StD)	1.00	1.00	1.00	0
Serajganj General Hospital (SgD)	0.85	1.00	0.85	− 1
Shahid Shamsuddin Hospital—Sylhet (SlD)	0.77	1.00	0.77	1
Shariatpur District Hospital (SpD)	1.00	1.00	1.00	0
Sherpur 100 bed District Sadar Hospital (SrD)	0.76	1.00	0.76	1
Sunamganj 250 bed District Sadar Hospital (SnD)	0.52	0.56	0.93	1
Tangail 250 bed District Hospital (TnD)	1.00	1.00	1.00	0
Thakurgaon District Hospital (TgD)	1.00	1.00	1.00	0
Mean	0.79	0.92	0.85	–
Median	0.83	1.00	0.92	–
Standard deviation	0.20	0.16	0.16	–
Minimum	0.36	0.48	0.36	–
Maximum	1.00	1.00	1.00	–

In terms of VRS technical efficiency, total 49 DHs (79%) were found to be efficient. The 13 inefficient DHs were on an average 63% efficient. The efficiency level of Bandarban district hospital was the lowest (36%) in VRS. Total 18 (29%) DHs were found to be scale-efficient in VRS model. Among the 44 scale-inefficient DHs, 31 (70.5%) had IRS, and 13 (29.6%) had DRS.

**Fig. 1:**
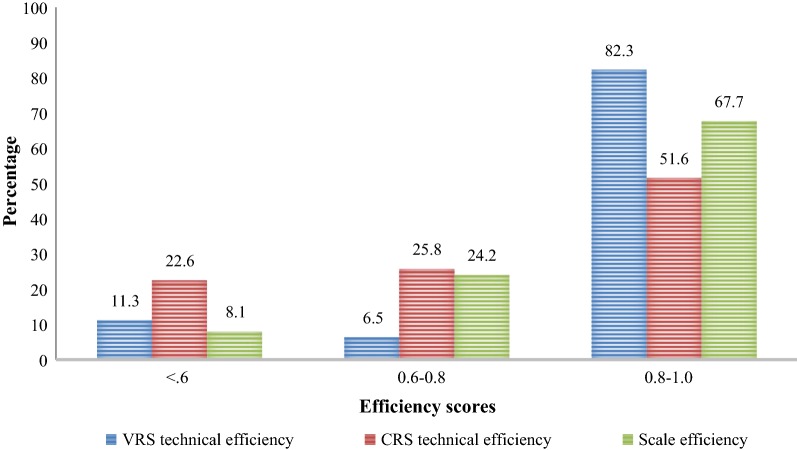
Percentage distribution of the district hospitals by technical (VRS and CRS) and scale efficiency scores

The majority of the DHs had all three types of efficiency scores (CRS technical efficiency, VRS technical efficiency, and scale efficiency) between 0.80 and 1.00 (Fig. 1). A few number of the DHs had VRS technical efficiency and scale efficiency less than 0.60.

Table 4 shows the mean efficiency scores of DHs by administrative divisions in Bangladesh. In Sylhet division, both CRS technical efficiency (0.71) and VRS technical efficiency (0.79) scores were the lowest among the administrative divisions. However, CRS technical efficiency score was the highest in Rangpur division, and VRS technical efficiency was the highest in Khulna division. The average scale efficiency score was the highest in Rajshahi division (0.94) and the lowest in Barisal division (0.78).

**Table 4:** Mean technical and scale efficiency scores of the district hospitals by division

Name of the divisions	Number of hospitals (N = 62)	Mean CRS technical efficiency score	Mean VRS technical efficiency score	Mean Scale efficiency score
Barisal	6	0.75	0.96	0.78
Chittagong	11	0.67	0.87	0.79
Dhaka	13	0.82	0.96	0.86
Khulna	10	0.83	0.97	0.85
Mymensingh	3	0.79	0.91	0.87
Rajshahi	7	0.85	0.90	0.94
Rangpur	8	0.87	0.94	0.93
Sylhet	4	0.71	0.79	0.90

The results of the Tobit regression model utilizing the CRS technical efficiency and VRS technical efficiency scores are presented in Table 5. The DHs with target population over 2.5 million had higher CRS technical efficiency compared to DHs with target population less than 1 million. The DHs located in a district with poverty headcount 15% to 30% had higher CRS technical efficiency and VRS technical efficiency than the DHs located in a district with poverty headcount less than 15%. Among the administrative division in Bangladesh, DHs in Chittagong, Dhaka, Khulna, Mymensingh had higher CRS efficiency than the DHs in Barisal. However, DHs in Sylhet division were less VRS technical efficient than the DHs in Barishal division. The coefficient was positive and statistically significant. As expected the CRS technical efficiency score of DHs increased with the increment of bed occupancy ratio and ratio to bed to physicians and nurses.

**Table 5:** Determinants of inefficiencies in district hospitals of Bangladesh

Variables	Coefficient (dependent variable = CRS technical inefficiency)	Coefficient (dependent variable = VRS technical inefficiency)
Population		
1 to 2.5 million (Ref ≤ 1 million)	− 0.117 (− 0.345, 0.111)	0.091 (− 0.122, 0.304)
Over 2.5 million (Ref ≤ 1 million)	− 0.238* (− 0.497, 0.021)	0.059 (− 0.183, 0.301)
Poverty headcount		
15% to 30% (Ref ≤ 15%)	− 0.422** (− 0.784, − 0.059)	− 0.376** (− 0.720, − 0.032)
30% > (Ref ≤ 15%)	− 0.256 (− 0.601, 0.090)	− 0.340** (− 0.669, − 0.011)
Bed occupancy ratio	− 0.637*** (− 0.813, − 0.462)	− 0.248*** (− 0.407, − 0.090)
Average length of stay	0.031 (− 0.016, 0.078)	0.026 (− 0.018, 0.070)
Ratio of beds to physicians	− 0.046*** (− 0.074, − 0.018)	− 0.006 (− 0.031, 0.020)
Ratio of beds to nurses	− 0.055** (− 0.109, − 0.000)	0.009 (− 0.043, 0.060)
Administrative division		
Chittagong (Ref = Barisal)	− 0.372** (− 0.718, − 0.027)	0.007 (− 0.288, 0.303)
Dhaka (Ref = Barisal)	− 0.442*** (− 0.761, − 0.124)	− 0.060 (− 0.335, 0.214)
Khulna (Ref = Barisal)	− 0.291* (− 0.619, 0.038)	0.059 (− 0.226, 0.345)
Mymensingh (Ref = Barisal)	− 0.242 (− 0.664, 0.180)	0.136 (− 0.243, 0.515)
Rajshahi (Ref = Barisal)	− 0.461** (− 0.818, − 0.105)	0.133 (− 0.182, 0.448)
Rangpur (Ref = Barisal)	− 0.248 (− 0.550, 0.054)	0.086 (− 0.186, 0.358)
Sylhet (Ref = Barisal)	0.022 (− 0.355, 0.399)	0.337* (− 0.010, 0.684)
Constant	2.363*** (1.602, 3.124)	0.616* (− 0.063, 1.294)
Sigma	0.256*** (0.205, 0.307)	0.244*** (0.198, 0.289)
N	62	62
Log-likelihood	− 10.74	− 3.845
Chi square (10)	56.71	23.32
p-value	0.000	0.08
R-square	0.730	0.752

* p < 0.10, ** p < 0.05, *** p < 0.01

## Discussion

The findings reflected that on an average the DHs were technically efficient. The average CRS technically efficiency was 79% and VRS technically efficiency was 92%, and therefore, using resources optimally. The level of technical efficiency is high because of the high utilization of DHs in Bangladesh. Another study using simple indicators of hospital performance and Pabon Lasso model, observed similar findings on the efficiency of DHs in Bangladesh [12]. The mean VRS and CRS technical efficiency score of the DHs may reflect that these DHs, on an average, could reduce their input mix by 8% and 21% respectively while maintaining the same level of output. However, most of these hospitals are functioning beyond their capacities since the average bed-occupancy rate of DHs was 137% (CI 121–147) in 2015 [3]. Therefore, there is a little scope for increase in efficiency gains in these hospitals by reducing inputs, and instead, additional investment on more beds and other accompanying resources will be required in order to improve outcomes. A study on the comparative efficiency of the healthcare facilities of Bangladesh showed that the DHs were more cost-efficient compared to the primary level Upazila health complexes [11].

Studies on the efficiency of the DHs conducted using DEA approach reported different findings in different settings. A study conducted in China found that only 8.8% of DHs were technically efficient in CRS technical efficiency and 15.8% DHs were technically efficient in VRS technical efficiency [20]. Another study conducted in Ghana found that out of 128 DHs, 31 (24%) were VRS technically efficient [27]. Two separate studies conducted in India reported that among the DHs studied, 50% were technically efficient in VRS technical efficiency [9, 28]. Thus, the available evidence indicated that technical efficiency scores vary among the DHs in Bangladesh, India, Ghana, Namibia, and China. This variation can be due to the associated environmental factors that influence the inputs and outputs of DHs of the countries.

The mean VRS technical efficiency scores were 0.92 and the CRS technical efficiency scores were 0.79 for DHs in Bangladesh. The findings of mean VRS technical efficiency score were similar to studies conducted in India (0.90) and Ghana (0.89) [9, 10]. A study in Namibia revealed that mean CRS technical efficiency was less than 75% [29].

Our study revealed that only 29% of the DHs were scale efficient meaning they operate at the required optimal size. The prevalent scale inefficiency was the highest (50%) in IRS. In the presence of IRS, expansion of output reduces unit cost. The hospital management does not require to increase the output level if there is no demand for that increased output [29]. However, increasing output level is not feasible in Bangladesh context since the over-capacity utilization already observed for DHs [3]. Therefore, the hospitals can be expanded to make them scale-efficient.

We extended the DEA analysis by using a Tobit model to identify influential factors that may lead to the existing inefficiency of the DHs. It was found that population size (over 2.5 million), poverty headcount, BOR, ALoS, RoBTP, and RoBTN had a significant influence on inefficiency regarding CRS technical efficiency scores. While population size, poverty headcount, BOR, and division had a significant influence on inefficiency regarding VRS technical efficiency. The result showed that using CRS technical efficiency, the DHs with large catchment population (over 2.5 million) had a lower chance of inefficiency than the DHs with the small catchment population (< 1 million). The possible reason could be the allocation of resources/inputs in DHs may not be according to catchment populations in Bangladesh. Therefore, there are possibilities of wastage in the hospitals with small catchment population since the DGHS is currently using bed size of the DHs as the main resource allocation criteria. The efficiency in resource allocation should be improved and contexualized by setting an input-mix formula for DHs considering health and socio-economic indicators (e.g., population density, poverty, bed occupancy ratio). The formula can be designed by learning from the input mix in the efficient DHs. The poverty headcount was negatively associated with inefficiency (p-value < 0.05) of the DHs which implied that such hospitals covering high poverty area were comparatively more efficient than the hospitals covering low poverty area. The findings could be attributable to the fact that public facilities (e.g., DHs) are more utilized by the poor people than the rich people in Bangladesh [30, 31]. The BOR was negative and significantly associated with technical inefficiency, indicating that technically inefficient DHs had a lower BOR. The RoBTN and RoBTP were negative and significantly associated with technical inefficiency which implied that the lower number of beds for each nurse or lower RoBTN were technically inefficient. However, the average RoBTN of the DHs was 2.94 which was higher than the developed countries (approximately 0.33) [32]. The DHs may operate using minimum human resource cost (i.e., higher RoBTP and higher RoBTN) to get higher efficiency, as it was found by the Tobit model. However, such minimum human resource allocation may affect the service quality of the DHs and consequently, this will affect the relationship between doctors and patients [20].

This study has several limitations. Firstly, the DEA approach, in general, cannot apprehend quality of health services. Secondly, we could not adjust the hospitals in terms of input–output mix therefore this may affect the interpretation for input variables reductions and expansions of the DHs. However, we studied DHs with similar case-mix that can address the issue for all DHs. Thirdly, due to the unavailability of information, several other input indicators such as the price of the drugs and the cost of the treatment were not included in this study. Since drugs, medical supplies, and equipment are procured centrally by the Central Medical Store Depot (CMSD) of the MoHFW, the prices may not vary widely across the DHs [33]. Therefore, drug price may not affect the estimated efficiency scores of the DHs. However, despite these limitations, this is the first study which estimates the technical and scale efficiency of the DHs of Bangladesh using multiple inputs and outputs variables.

## Conclusion

Health managers or policymakers need information about how well the DHs are utilizing the available resources to improve the performance of DHs in Bangladesh. Using routinely available data, this study shed light on the efficiency of the DHs applying DEA to understand the comparable score across the facilities. The findings of this efficiency study provided empirical evidence on the efficiency level of public DHs in Bangladesh and associated institutional and environmental factors. The average efficiency score of the inefficient DHs was 63% (13 of 62 in VRS technical efficiency) and 70% (44 of 62 in CRS technical efficiency). These DHs would need to improve their performance. The higher technical efficiency of the DHs is likely to facilitate better utilization of resources, control the cost of medical services, and consequently to provide more affordable healthcare. The policymakers and hospital managers can use the efficiency estimate of this study to promote benchmarking among the DHs where inefficient DHs can learn from efficient DHs. The MoHFW can set input mix for DHs considering different important resource allocation factors (e.g., population density, poverty) to avoid inefficiency. The DHs at having a maximum and minimum level of efficiency should be investigated further to understand how and why the services provision systems are operating differently at these DHs. Further studies can be conducted to explore the causes of inefficiency in DHs. The policymakers can develop context-based strategies for the inefficient DHs to improve their efficiency in delivering healthcare which may be useful to address the unmet need for healthcare services in Bangladesh.

## Data Availability

The datasets used during the current study are available at MIS Local Health Bulletin of DGHS. http://app.dghs.gov.bd/localhealthBulletin2015/publish/.

## Abbreviations

ALoS: Average length of stay
ANC: Antenatal care
BBS: Bangladesh Bureau of Statistics
BCC: Banker, Charnes, and Cooper
BOR: Bed occupancy ratio
CCR: Charnes, Cooper, and Rhodes
CMSD: Central Medical Store Depot
CRS: Constant returns to scale
DEA: Data envelopment analysis
DGHS: Directorate General of Health Services
DHs: District hospitals
DMUs: Decision making units
DRS: Decreasing returns to scale
IRS: Increasing returns to scale
MoHFW: Ministry of Health and Family Welfare
OPD: Outpatient department
PNC: Post-natal care
RoBTN: Ratio of beds to nurse
RoBTP: Ratio of beds to physicians
SD: Standard deviation
SDG: Sustainable development goals
USD: United States Dollar
VRS: Variable returns to scale
